# A Heart Under Pressure: Anesthetic Management of a Large Ascending Aortic Aneurysm Mimicking Mediastinal Mass Physiology

**DOI:** 10.1155/cria/6474734

**Published:** 2026-06-20

**Authors:** Geronimo Garcia, Ermias Araia, Brian Frondorf, Shyamal R. Asher

**Affiliations:** ^1^ Department of Anesthesiology, Brown University Health, Providence, Rhode Island, USA; ^2^ Department of Anesthesiology, Boston Medical Center, Boston, Massachusetts, USA, bu.edu

**Keywords:** ascending aortic aneurysm (AscAA), mediastinal mass, transesophageal echocardiography (TEE)

## Abstract

Large ascending aortic aneurysms (AscAAs), defined as exceeding 8 cm, are exceedingly rare and carry high mortality due to the combined risks of rupture and mediastinal compression. Their management demands precise anesthetic, surgical, and perfusion coordination to prevent hemodynamic collapse and neurologic injury. We report an 83‐year‐old woman with an 8.9 × 7.7 cm AscAA producing marked right ventricular and pulmonary artery compression. Perioperative management emphasized advanced monitoring, preservation of venous return during induction, and readiness for immediate cardiopulmonary bypass (CPB). Given the aneurysm’s proximity to the sternum and high rupture risk, CPB via right axillary cannulation was established prior to sternotomy, followed by deep hypothermic circulatory arrest and complex aortic reconstruction. Intraoperative transesophageal echocardiography (TEE)–guided cannulation assessed ventricular filling under dynamic compression and confirmed graft and valve function. The patient recovered without major complications. This case underscores the physiologic overlap between giant aneurysms and anterior mediastinal masses, where loss of spontaneous ventilation or preload reduction may precipitate cardiovascular collapse. Our report additionally provides multimodal imaging that demonstrates the degree of anatomic distortion caused by a giant AscAA and includes a concise review of previously published cases. Effective management of these rare, life‐threatening aneurysms relies on anticipating dynamic obstruction physiology, preparing for rapid bypass initiation, and maintaining continuous interdisciplinary coordination throughout the perioperative course.

## 1. Introduction

Thoracic aortic aneurysms represent a spectrum of life‐threatening vascular dilations that predispose patients to rupture, dissection, and sudden cardiovascular collapse [1]. The ascending aorta is the most common site, accounting for approximately 60% of all thoracic aortic aneurysms, with overall prevalence estimated at 5–10 cases per 100,000 person‐years and rising incidence in elderly populations due to improved imaging and longevity [1]. While most aneurysms are diagnosed at diameters between 5 and 6 cm, large ascending aortic aneurysms (AscAAs), defined as those exceeding 8 cm, are exceedingly rare and comprise less than 0.03 percent of thoracic aneurysms reported in the literature [2, 3]. At these sizes, the annual risk of rupture or dissection approaches 30–40%, with untreated mortality exceeding 50–60% [1, 2].

The pathophysiology of AscAAs reflects progressive medial degeneration characterized by loss of smooth muscle cells, fragmentation of elastic fibers, and accumulation of proteoglycans, a process termed cystic medial necrosis [1]. Genetic predispositions such as Marfan syndrome, Loeys–Dietz syndrome, Ehlers–Danlos syndrome, and bicuspid aortic valve can accelerate aortic wall weakening, though the majority of cases in elderly patients are degenerative and hypertension‐related. Chronic hemodynamic stress contributes to ascending aortic wall remodeling, dilation, and eventual aneurysm formation [1].

From an anesthetic standpoint, giant AscAAs pose the dual physiologic hazards of catastrophic rupture and compressive effects on adjacent mediastinal structures, particularly the right ventricle, pulmonary artery, and superior vena cava [2, 4]. These dynamics resemble mediastinal mass physiology, where loss of spontaneous ventilation or abrupt intrathoracic pressure changes can precipitate cardiovascular collapse [5, 6]. The perioperative plan must therefore balance minimizing aortic wall stress with preserving venous return and right ventricular filling. This requires tailored induction techniques, vigilant hemodynamic monitoring, and readiness for immediate cardiopulmonary bypass (CPB) [3, 5, 6].

The following case describes an 83‐year‐old woman with a massive ascending and proximal arch aneurysm measuring 8.9 by 7.7 cm, producing significant right ventricular and pulmonary artery compression. Her management emphasizes multidisciplinary coordination, advanced monitoring, transesophageal echocardiography (TEE) guidance, and the use of deep hypothermic circulatory arrest (DHCA) with antegrade cerebral perfusion [3, 7–9].

## 2. Case Presentation

An 83‐year‐old woman with hypertension and polymyalgia rheumatica presented with several months of progressive dyspnea on exertion. She denied chest pain, connective tissue disease, trauma, or prior cardiac surgery. Computed tomography (CT) of the chest revealed an 8.9 cm by 7.7 cm ascending aortic and proximal arch aneurysm with compression of the right ventricle and main pulmonary artery (Figure 1). Preoperative transthoracic echocardiography (TTE) demonstrated normal left ventricular systolic function with an ejection fraction of 55–60%, normal right ventricular function, severe aortic and severe mitral regurgitation, a moderate pericardial effusion and a large AscAAs. After shared decision‐making, the patient and her family elected to proceed with operative repair.

**FIGURE 1:**
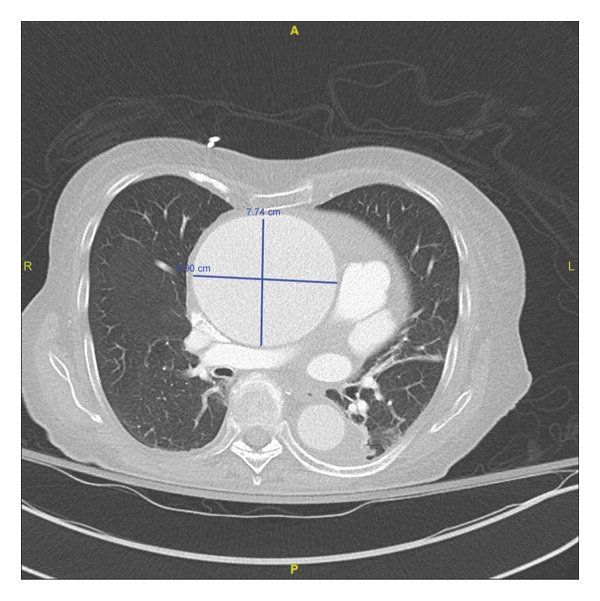
CT scan of the chest showing extremely large 8.9 × 7.7 cm ascending aortic aneurysm extending into the aortic arch.

Standard American Society of Anesthesiologists monitors and advanced hemodynamic monitoring were applied, including right radial and left femoral arterial lines, a right internal jugular introducer with pulmonary artery catheter, a central venous catheter, a nasopharyngeal temperature probe, and a TEE probe. Prebypass TEE confirmed the preoperative TTE findings, including a large aortic aneurysm distorting the cardiac anatomy (Figures 2 and 3) and compressing the pulmonary artery (Figure 4).

**FIGURE 2:**
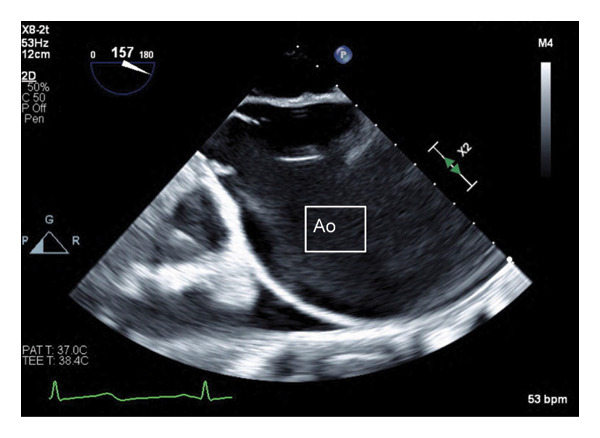
Intraoperative TEE image with mid‐esophageal long axis view showing a large ascending aortic aneurysm and compression of the surrounding structures.

**FIGURE 3:**
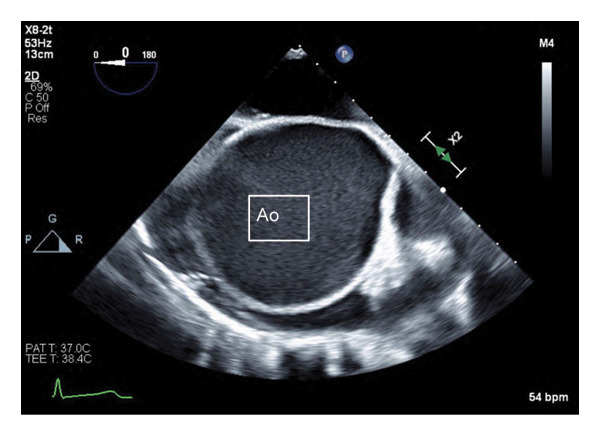
Intraoperative TEE image with mid‐esophageal ascending aorta short‐axis view showing a large ascending aortic aneurysm displacing other structures of the heart.

**FIGURE 4:**
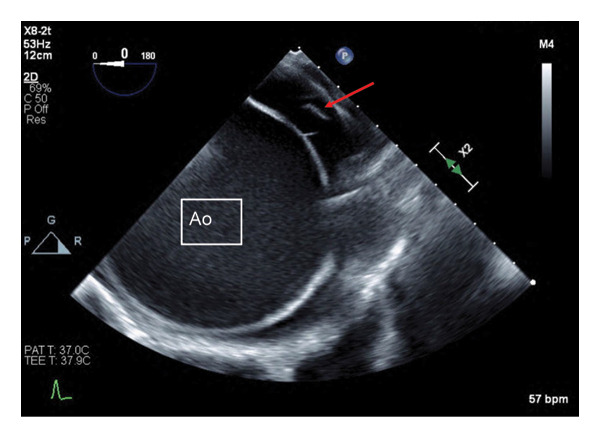
Intraoperative TEE image with ascending aortic short‐axis view showing a large ascending aortic aneurysm with compression of the pulmonary artery and the tip of the pulmonary artery catheter in view (red arrow).

Induction of general anesthesia was performed using a carefully controlled, hemodynamically focused approach designed to minimize the risk of cardiovascular collapse from dynamic compressive physiology. Given the significant right ventricular and pulmonary artery compression observed on preoperative imaging and TEE, the anesthetic plan prioritized preservation of preload, avoidance of abrupt reductions in systemic vascular resistance, and minimization of sympathetic surges that could increase aortic wall stress. Standard preoxygenation was performed, and anesthetic agents were administered in a gradual, titrated fashion using midazolam 2 mg, fentanyl 4 mcg/kg, and incremental doses of propofol up to 1 mg/kg. An esmolol infusion at 300 mcg/kg/min was utilized during induction to blunt hypertensive responses and reduce shear stress across the aneurysmal aortic wall. Positive pressure ventilation was introduced cautiously following airway securement to avoid sudden impairment of venous return and worsening right‐sided compression physiology. Given the high risk of catastrophic decompensation or rupture during induction, the CPB team remained immediately available with perfusion equipment primed and prepared for rapid initiation of bypass if needed.

Given the aneurysm’s large size and anterior displacement, the surgical team elected to initiate CPB via right axillary artery cannulation prior to sternotomy. Therefore, a right radial arterial line was placed to monitor blood pressure downstream of the aortic cannula, and the femoral arterial line would allow measurement of systemic blood pressure. A right infraclavicular cutdown facilitated anastomosis of an 8 mm graft to the axillary artery for antegrade cerebral perfusion. A right femoral venous cutdown was used for placement of a multistage venous drainage cannula under TEE guidance. After adequate anticoagulation, CPB was initiated, and the patient was cooled to 18 degrees Celsius.

Sternotomy revealed an enormous aneurysm displacing the heart inferiorly (Figure 5). The pericardium contained a moderate serous effusion that was evacuated. The aneurysmal aorta was resected and reconstructed with a 36‐mm Gelweave graft, and the native trileaflet aortic valve was replaced with a 23‐mm INSPIRIS bioprosthetic valve. During DHCA, antegrade cerebral perfusion was provided through the axillary graft. The innominate and left common carotid arteries were reimplanted into the graft before controlled rewarming. The total CPB time was 313 min, and the total cross‐clamp time was 219 min.

**FIGURE 5:**
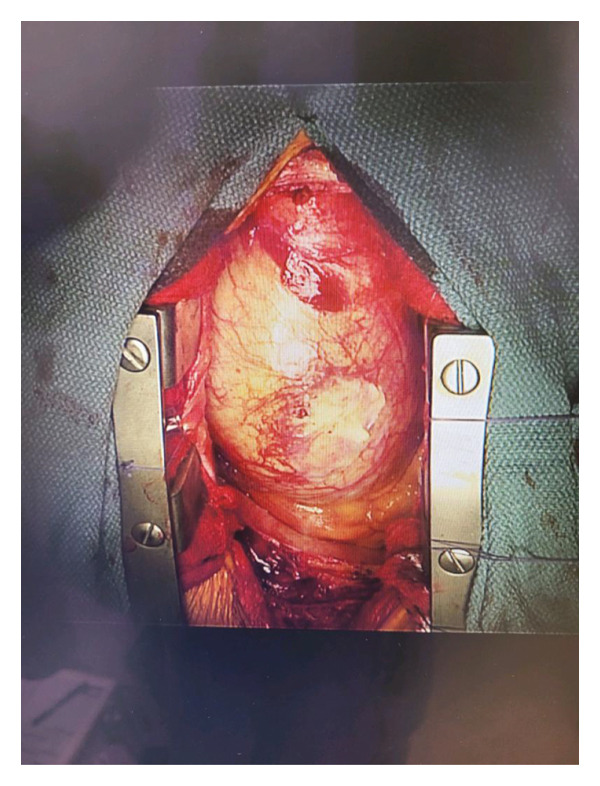
Intraoperative picture showing a large ascending aortic aneurysm dominating the surgical field.

The patient was successfully weaned from CPB with careful vasoactive support tailored to maintain coronary perfusion, right ventricular function, and systemic perfusion pressure during rewarming and postbypass myocardial recovery. Norepinephrine at 4 mcg/min and epinephrine at 2 mcg/min were initiated to support mean arterial pressures between 65 and 75 mm Hg while maintaining adequate cardiac output and ventricular filling. Hemodynamic management throughout cooling, circulatory arrest, and rewarming relied on continuous assessment using dual arterial pressure monitoring, pulmonary artery catheterization, serial arterial blood gas analysis, and TEE. Particular attention was directed toward avoiding excessive vasodilation, maintaining adequate preload, and minimizing abrupt changes in afterload that could compromise myocardial performance following prolonged bypass and DHCA.

Postprocedure TEE revealed normal biventricular systolic function without regional wall motion abnormalities. A well‐seated bioprosthetic aortic valve exhibited normal leaflet motion with no paravalvular or central regurgitation and low transvalvular gradients (peak/mean 12/6 mmHg). Mitral regurgitation improved to mild, with no other significant valvular abnormalities. The patient was resuscitated with blood products, including 2 units of cryoprecipitate, 2 units of platelets, 2 units of packed red blood cells, and 4 units of fresh frozen plasma, and 395 mL was returned via Cell Saver processing. KCentra was administered at approximately 32.5 units/kg.

The patient was transferred to the cardiothoracic intensive care unit, sedated and hemodynamically stable. She was extubated on Postoperative day 3 without neurologic deficits or major complications.

Informed consent for surgical intervention, anesthetic management, and publication of this de‐identified case was obtained from the patient.

## 3. Discussion

Large AscAAs exceeding 8 cm are an exceptionally rare presentation of thoracic aortic disease and pose unique challenges to both anesthesiologists and surgeons [1, 2]. In this case, the aneurysm’s 9 cm diameter and direct compression of the right ventricle and pulmonary artery created a physiology analogous to an anterior mediastinal mass [2, 4]. During induction, loss of spontaneous ventilation and systemic vasodilation could have precipitated catastrophic venous return obstruction and cardiovascular collapse [5, 6]. Recognition of this risk shaped a carefully controlled induction strategy emphasizing preservation of preload, avoidance of abrupt hemodynamic shifts, cautious introduction of positive pressure ventilation, and immediate readiness for CPB in the event of cardiovascular collapse, principles similarly described in the anesthetic management of anterior mediastinal mass physiology and large thoracic aneurysms with compressive features [4–6].

Given the aneurysm’s size, posterior sternal proximity, and compressive effects on the right ventricle and pulmonary artery, presternotomy CPB via right axillary artery cannulation was established to facilitate controlled circulatory support prior to sternotomy and to reduce the risk of catastrophic hemorrhage during chest entry [3, 8, 9]. DHCA with antegrade cerebral perfusion was utilized to facilitate complex aortic repair while minimizing neurologic injury risk. The anesthetic and surgical strategy incorporated several contemporary neuroprotective principles, including multimodal invasive monitoring, transesophageal echocardiographic assessment, controlled hypothermia, embolic‐risk reduction, careful vasoactive management, and cerebral perfusion optimization throughout cooling and rewarming phases [8–12]. Modern neuroprotection during complex aortic surgery increasingly emphasizes coordinated perioperative management rather than reliance on deep hypothermia alone, although substantial institutional variability persists regarding optimal neuroprotective protocols and perfusion targets during circulatory arrest [10–12].

The anesthetic management required dynamic balancing of preload, afterload, and contractility throughout cooling, arrest, and rewarming phases [8, 9, 13]. Comprehensive multimodal monitoring, including dual arterial lines, pulmonary artery catheterization, invasive venous access, and continuous TEE assessment, enabled rapid titration of vasoactive infusions and guided perfusion management throughout cooling, circulatory arrest, and rewarming phases [5, 6, 8, 9, 13, 14]. Careful balancing of preload, afterload, and contractility was required to maintain systemic perfusion while minimizing myocardial stress and postoperative bleeding risk during prolonged bypass and circulatory arrest.

From a surgical perspective, the choice to repair both the ascending aorta and proximal arch using a Gelweave graft, coupled with an INSPIRIS prosthetic valve, reflected the multidisciplinary team’s assessment that the aneurysm extended beyond the sinotubular junction and that the aortic valve leaflets were structurally unsalvageable [2]. The improvement in moderate to mild mitral regurgitation following correction of aortic regurgitation and relief of right ventricular compression confirmed the functional, rather than structural, nature of the mitral pathology and underscored how comprehensive pre‐ and intraoperative echocardiographic evaluation directly informs surgical decision‐making [7].

This case exemplifies the importance of multidisciplinary coordination between anesthesiology, surgery, perfusion, and critical care teams. Preoperative imaging and TEE assessment guided cannulation and bypass strategy, anesthetic planning prioritized hemodynamic control, and surgical technique minimized ischemic and neurologic injury [5–9]. Each stage of management required anticipation of collapse physiology and adaptation of mediastinal mass principles to the aortic domain [4–6].

Published reports of giant AscAAs consistently highlight the dual hazards of rupture and compressive physiology affecting the right heart and great vessels [2, 4, 15–18] (Table 1). Several case reports describe severe pulmonary artery or right ventricular compression leading to pulmonary hypertension or right‐sided failure, including cases of Felix et al. and Abe et al. [4, 17]. Superior vena cava syndrome has been reported in extremely large aneurysms, such as in the series by Bicer et al. [2]. Shah et al. described a giant AscAA characterized using multimodality imaging, underscoring the importance of carefully controlled induction, cautious transition to positive pressure ventilation, and preparation for rapid institution of CPB [15]. Additional reports highlight unusual presentations such as saccular aneurysm following valve replacement or right pulmonary artery compression leading to right heart failure, illustrating the diverse ways mass‐effect physiology may manifest [16, 17]. A recent case report by Dumani et al. detailed a giant ascending aortic and arch aneurysm requiring extensive reconstruction, reinforcing the complexity of surgical planning in such cases [18]. Preinduction or peri‐induction hemodynamic collapse has been reported in large unruptured thoracic aneurysms, underscoring the importance of carefully controlled induction, cautious transition to positive pressure ventilation, and preparation for rapid institution of CPB [5, 6].

**TABLE 1:** Comparison of recent large ascending aortic aneurysm (AscAA) case reports.

Case report	Age (years)/sex	Max diameter (cm)	Compression finding	Outcome/notes
Bicer et al. [2]	77/Female	14.0	SVC syndrome; large AscAA	Largest reported AscAA; surgical replacement
Felix et al. [4]	32/Male	10.3	Severe PH from PA compression	Right heart strain; emphasizes compression physiology
Inoue et al. [5]	32/Male	10.0	Airway/PA compression physiology	Peri‐induction collapse with GA; cautionary
Shah et al. [12]	73/Male	10.0	Mass effect; calcified AscAA	Successful surgical repair
Park et al. [13]	58/Male	4.56	RPA compression post‐AVR	Unusual saccular aneurysm; prior BAV/AVR
Abe et al. [14]	73/Female	6.7	RPA compression ⟶ RHF	Right heart failure secondary to PA compression
Dumani et al. [15]	40/Male	13.0	Giant AscAA + arch	Arch involvement; surgical replacement

Abbreviations: AscAA, ascending aortic aneurysm; AVR, aorta valve replacement; BAV, bicuspid aortic valve; GA, general anesthesia; PA, pulmonary artery; PH, pulmonary hypertension; RHF, right heart failure; RPA, right pulmonary artery; SVC, superior vena cava.

With respect to cannulation and neurologic protection strategies, axillary artery cannulation is widely reported as a favorable site in ascending aortic and arch surgery for establishing antegrade cerebral perfusion and minimizing embolic risk [8, 9, 15, 18]. Neuroprotection strategies during arch repair increasingly favor DHCA coupled with antegrade cerebral perfusion, which clinical series and reviews associated with improved neurologic outcomes compared with DHCA alone [8, 9, 15, 18].

Our case aligns with prior reports in several ways: the presence of compressive physiology involving the right ventricle and pulmonary artery [2, 4, 15, 17, 18], the use of axillary cannulation permitting antegrade cerebral perfusion [8, 9, 15, 18], the use of DHCA for safe arch reconstruction [8, 9, 15, 18], and reliance on continuous TEE for dynamic assessment and surgical decision‐making [7, 15, 18].

This case also adds new teaching points. The patient’s advanced age is at the upper extreme of reported cohorts while maintaining preserved biventricular function [2, 4, 15, 17, 18]. CPB initiation prior to sternotomy due to aneurysm proximity to the posterior sternal table represents a risk‐mitigation strategy not uniformly reported in similar cases [8, 9, 15, 18]. Multimodal vasoactive management was titrated to defined arterial pressure targets while utilizing a carefully controlled induction strategy prior to CPB readiness [5, 6]. Concomitant aortic valve replacement with improvement of functional mitral regurgitation demonstrates the interplay between valve pathology and mass‐effect physiology [7, 15].

## 4. Conclusions

Giant AscAAs are uncommon and are most often characterized in the literature through individual case reports. These aneurysms may produce both rupture risk and compressive physiology, creating significant challenges for perioperative planning and cardiopulmonary stability. This report contributes an additional example of this rare pathology, emphasizing the anesthetic and surgical considerations required for safe management and illustrating the value of multimodality imaging in defining anatomy and guiding operative strategy. The case highlights principles relevant to anesthesiologists managing patients with dynamic mediastinal mass–like physiology. A concise overview of prior case reports, associated imaging approaches, and operative interventions is provided for comparative context.

## Funding

No funding was received for this study.

## Conflicts of Interest

The authors declare no conflicts of interest.
